# 

**Published:** 1889-06

**Authors:** 


### The Chicago Medical Journal and Examiner.

Volume 58. Number 6.

### CONTENTS OF THIS NUMBER.

**Original Communications.** PAGE

Mechanical Treatment of Hip-joint Disease. W. Blanchard .......... 321
Torsion of Arteries for the Arrest of Hæmorrhage. J. B. Murdoch .......... 325

**Extracts and Abstracts** .......... 327

**Editorial—**

Toxic Effect of Antipyrine .......... 339

**Society Reports—**

Illinois State Medical Society. Thirty-Ninth Annual Meeting .......... 340
Gynæcological and Obstetrical Society of Baltimore. Stated Meeting, March 12 .......... 349
National Association of Railway Surgeons. Second Annual Meeting .......... 355
New York Academy of Medicine. Section of Neurology. Stated Meeting, April 12 .......... 359
British Gynæcological Society. Meeting, April 10 .......... 361
The Medical Society of London. The Annual Conversazione .......... 365
The Clinical Society of London .......... 368
Hunterian Society of London .......... 373
British Gynæcological Society .......... 373

**Book Reviews** .......... 374

**Books Received** .......... 377

**Miscellany** .......... 378

